# Phenotypic and genotypic characterization of carbapenem resistant *Klebsiella pneumoniae* clinical isolates from Damietta, Egypt

**DOI:** 10.1186/s12866-026-04797-z

**Published:** 2026-02-27

**Authors:** Mohamed I. Abou-Dobara, Ahmed K. A. El-Sayed, Radwa N. Elmaghalawy, Hazem H. Saleh

**Affiliations:** 1 Botany and Microbiology Department, Faculty of Science, Damietta University, New Damietta, 34517 Egypt; 2 Urology and Nephrology Center, Mansoura University, Mansoura, Egypt

**Keywords:** Carbapenem-resistant *Klebsiella pneumoniae*, *blaNDM*, *blaOXA-48*, Virulence factors, Biofilm, Colibactin

## Abstract

**Background:**

The notable emergence of carbapenemase production is a critical problem threatening human health worldwide. Hence, this study aimed to monitor the prevalence of carbapenemase-producing *Klebsiella pneumoniae* in Damietta, Egypt. Additionally, the study investigated the association of carbapenemase producers and certain virulence traits, including biofilm formation and colibactin genotoxin.

**Methods:**

This study included 86 clinical isolates of *Klebsiella pneumoniae* collected from various clinical analytical laboratories and private clinics in Damietta, Egypt, between June 2023 and May 2024. Carbapenemase production was determined by various phenotypic assays (CarbaNP/mCIM/eCIM). Genotypic detection of carbapenemase-encoding genes (*blaNDM*,* blaKPC*,* blaVIM*,* blaIMP*, and *blaOXA-48*) and certain virulence genes (*clbA*, *clbB*, *mrkD*, and *fimH*) was performed using multiplex PCR. Chi-square and Fisher’s exact tests were used for statistical analysis.

**Results:**

In the current study, 44.2% (38/86) of *Klebsiella pneumoniae* were identified as carbapenemase producers. All carbapenemase-producing *Klebsiella pneumoniae* (38/38, 100%) were positive by mCIM, while 92.1% (35/38) were positive by CarbaNP, and 65.8% (25/38) were positive by eCIM. The *blaNDM*, *blaOXA-48*, and *blaKPC* genes were detected in 71%, 36.8%, and 5.2% of the tested isolates, respectively. No *blaIMP* or *blaVIM* were detected. Different biofilm formation capabilities were observed among carbapenemase producers: moderate (42.1%), strong (36.8%), weak (13.1%), and non-biofilm (7.8%). The capacity for biofilm formation differed significantly between carbapenemase-producing and non-producing *Klebsiella pneumoniae*. The *mrkD* gene was significantly associated with carbapenemase-producing *Klebsiella pneumoniae* in contrast to *fimH* and colibactin-encoding genes.

**Conclusions:**

This study provides valuable insights into the continuous emergence of carbapenemase-producing *Klebsiella pneumoniae*, which poses a serious clinical and public health concern. A significant association was observed between carbapenemase producers and certain virulence factors. This emphasizes the urgent need for ongoing monitoring, effective stewardship programs, intensive surveillance, and strict infection control policies to control the spread of such highly resistant strains.

**Supplementary Information:**

The online version contains supplementary material available at 10.1186/s12866-026-04797-z.

## Background

The dramatic rise of antimicrobial resistance (AMR) is considered one of the significant challenges threatening the healthcare system worldwide. Specialists of the World Health Organization (WHO) expect that the incidence rate of infectious diseases by 2050 will be the same as in the “pre-antibiotic era” due to the ongoing acquisition of antibiotic resistance [1]. The emergence of AMR is a serious risk that affects not only global health but also has harmful impacts on other aspects of life, such as food security and the economy, hindering progress in achieving Sustainable Development Goals 2030. The extensive use of antibiotics has resulted in the acquisition of drug resistance to a wide range of available antibiotics. Consequently, the isolates exhibited production of the extended-spectrum β-lactamases (ESBLs), which show high resistance against penicillins, cephalosporins, and monobactams [2]. Therefore, treatment of these multidrug-resistant (MDR) bacteria has relied on carbapenems, which have proven to be effective against complicated hospital and community-acquired infections caused by ESBLs and have served as the last resort of therapy [3]. Unfortunately, carbapenemase production has rapidly emerged at an alarming rate, especially among Enterobacteriaceae [4]. In addition, it is implicated as one of the key reasons causing the high rates of morbidity and mortality worldwide [5].

The carbapenem-resistant *Klebsiella pneumoniae* (CR-Kp) is considered the most common member among carbapenem-resistant Enterobacteriaceae worldwide [6]. The spread of carbapenemase-producing *Klebsiella pneumoniae* (CP-Kp) has been critically observed in low and middle-income countries (LMICs), including Egypt [7]. Carbapenemase-hydrolyzing enzymes are divided into three classes: A, B, and C [8]. Additionally, carbapenemases are classified into two subgroups based on the divalent cations in their active sites: the first group includes serine carbapenemases (classes A and D), and the second includes metallo-β-lactamases (MBLs, zinc carbapenemase) represented by class B. The most common carbapenemases among Enterobacteriaceae are *K. pneumoniae* carbapenemase (KPC) and oxacillinases such as OXA-48 enzymes, in addition to MBLs, which include imipenemase (IMP), New Delhi metallo-β-lactamase (NDM), and Verona integron-encoded metallo-β-lactamase (VIM) [9].

The survival strategy of MDR *K. pneumoniae* within the host cells is not enhanced solely through the acquisition of antibiotic resistance; it also requires certain virulence traits. The most significant trait in *K. pneumoniae* linked to its high pathogenicity is biofilm formation [10]. Furthermore, biofilm-associated infections are more difficult to treat than planktonic bacteria due to the acquisition of high antibiotic resistance and their antiphagocytic character, which helps in evading the host immune system [11, 12]. Specific adhesive organelles called fimbriae promote biofilm formation as type 3 and type 1 fimbrial adhesins, which are encoded by *mrkD* and *fimH* genes, respectively [13, 14].

Moreover, some pathogenic bacteria are capable of producing a wide range of toxins with cytogenic or cytotoxic effects, which enables them to survive within host cells [15]. In 2006, a polyketide synthase (PKS) genomic island (*clbA-clbS*) was first identified in *Escherichia coli*, which is involved in colibactin biosynthesis [16]. Later, it was also found in other members of Enterobacteriaceae, such as *K. pneumoniae* [17]. Colibactin is a peptide-polyketide hybrid genotoxin that has a remarkable role in pathogenicity in eukaryotes. It induces DNA double-strand breaks, which are associated with various clinical symptoms, such as cell cycle arrest, megalocytosis, gene mutations that promote tumor development, and ultimately cell death [18, 19].

The present study was conducted to monitor the emergence of carbapenemase-producing *K. pneumoniae* as the first epidemiological study on their prevalence in the Damietta Governorate. Additionally, due to the little literature on the prevalence of colibactin genotoxin in Egypt, we also aimed to evaluate its occurrence among CP-Kp isolates. Furthermore, the study investigates the association between carbapenemase producers and certain virulence factors.

## Methods

### The bacterial isolation and identification

A total of 86 clinical isolates of *K. pneumoniae* were collected from various clinical analytical laboratories and private clinics in Damietta, Egypt, between June 2023 and May 2024 under strict aseptic conditions. The clinical specimens were obtained within 12 h of collection from various clinical sources: midstream urine (38/86), sputum (29/86), and wound swab (19/86).

Samples underwent routine microbiological identification [20] via Gram stain and biochemical tests. For further identification, the isolates were inoculated on different culture media such as Blood agar, MacConkey agar, and Eosin Methylene Blue (EMB) agar (Oxoid Ltd., England). The specimens of urine were primarily cultured on Cysteine Lactose Electrolyte Deficient (CLED, Oxoid Ltd., England), and the bacterial growth that exceeds 10^5^CFU/mL will be processed for further identification [21]. According to the morphological properties of the bacterial colony, all the expected *Klebsiella* strains were confirmed up to the species level using the automated Vitek 2 system (BioMérieux, Marcy-l’Étoile, France).

### Antimicrobial susceptibility test

To demonstrate the antimicrobial susceptibility of *K. pneumoniae* isolates, the Kirby-Bauer method was applied [22]. Each bacterial suspension, adjusted to a turbidity of 0.5 MacFarland Standard (1.5 × 10^8^ CFU/ml ), was cultured on Muller-Hinton agar (MHA) plates (Oxoid Ltd., England). The antimicrobial susceptibility was assessed using the following antibiotic discs: amoxicillin/clavulanate (20/10 µg), ampicillin (30 µg), cefotaxime (30 µg), ceftazidime (30 µg), cefepime (30 µg), chloramphenicol (30 µg), doxycycline (30 µg), levofloxacin (5 µg), nalidixic acid (30 µg), gentamicin (10 µg), tobramycin (10 µg), meropenem (10 µg), and imipenem (10 µg). After incubation, the diameter of the inhibition zone was recorded, and the results were interpreted according to Clinical and Laboratory Standards Institute (CLSI) breakpoints [23]. All isolates that exhibited a resistant pattern to meropenem or imipenem were classified as carbapenem-resistant.

### Quantitative biofilm formation assay

The tissue culture plate (TCP) method was applied to quantify biofilm formation, as previously described by Stepanović et al. [24]. The bacterial isolates were grown overnight in tryptic soya broth (TSB, Oxoid Ltd., England) supplemented with glucose (1%). Then, each bacterial suspension was diluted 1:100 in the same medium. 200 µl of each diluted suspension was inoculated into a sterile flat-bottomed 96-well polystyrene microtiter plate, followed by incubation at 37℃ for 24 h without agitation. After that, each well was gently washed three times with 300 µl of phosphate buffer saline (PBS, pH 7.2), fixed by adding 150 µl of 99% methanol to each well for 20 min, and then 150 µl of crystal violet (2%) was added for staining the adherent cells for 15 min. Finally, resolubilized the stain with 150 µl of ethanol (95%). An uninoculated medium was employed as a negative control. The absorbance of the wells was measured at 570 nm using a microtiter-plate reader. The cut-off optical density was determined as three standard deviations (SD) above the mean OD of the negative control. The clinical isolates and controls were performed in triplicate. The results were categorized as follows:

- Strong biofilm production (4×OD_control< OD_sample).
- Moderate biofilm production (2×OD_control< OD_sample ≤ 4×OD_control).
- Weak biofilm production (OD_control< OD_sample ≤ 2×OD_control).
- Non-biofilm production (OD_sample≤OD_control).

### The phenotypic detection of carbapenemase production

#### Colorimetric microtube assay (CarbaNP test)

1 µl of an overnight culture of the tested isolates was added to two Eppendorf tubes (A and B), each containing 100 µl of lysis buffer (20 mM Tris-HCl). Then, 100 µl of solution A (phenol red solution, pH 7.8, containing 0.1 mM ZnSo_4_) was added to tube A. While tube B contained 100 µl of solution A supplemented with 12 mg/ml of imipenem/cilastatin. Both tubes were finally incubated for 2 h at 37℃ and examined visually to detect any color change. Positive carbapenemase producers were detected via the color change in tube B from red to yellow, dark yellow, or light orange [25].

#### The mCIM and eCIM test

A loopful (1 µl) of an overnight culture was suspended in a 2 ml sterile TSB media [25]. While another 1 µl of the tested isolate was suspended in 2 ml TSB media supplemented with 20µL of ethylenediaminetetraacetic acid (0.5 mM EDTA). Then, 10 µg of meropenem disc (MEM) was added to both tubes and incubated for 4 h at 37 °C. After incubation, the discs were applied to MHA plates, which were inoculated with a suspension of carbapenem-susceptible *E. coli* ATCC 25,922 as an indicator strain, and incubated for 24 h at 37 °C. The resulting data were interpreted as shown in Table 1 according to CLSI [23].

**Table 1:** Interpretation of the mCIM/eCIM test according to CLSI M100, 33rd ed. (2023)

mCIM	Carbapenemase positive: isolates with a zone of inhibition of 6–15 mm or 16–18 mm if pinpoint colonies were present within the inhibition zone.
	Carbapenemase negative: isolates with a zone diameter ≥ 19 mm (clear zone).
eCIM	Metallo-β-lactamase (MBL) production: The increase in the zone of inhibition was ≥ 5 mm compared to the zone diameter recorded in mCIM.
	Serine production: The increase in zone diameter was ≤ 4 mm compared to the zone of inhibition in mCIM.

### Chromosomal DNA extraction

The phenol/chloroform method was applied to extract the chromosomal DNA according to [26]. The DNA was stored at -20℃ until needed.

### Molecular detection of virulence and carbapenemase-encoding genes

Two distinct multiplex PCR assays were employed to investigate the presence of virulence and carbapenemase-encoding genes among *K. pneumoniae* isolates. The used primers and the cycling conditions are listed in Table 2. The first multiplex PCR reaction was amplified for targeting the colibactin-encoding genes (*clbA* and *clbB* genes) and biofilm-related genes (*mrkD* and *fimH* genes). The second reaction was carried out to detect the carbapenemase-encoding genes (*blaNDM*,* blaKPC*,* blaVIM*,* blaIMP*, and *blaOXA-48*). The PCR reactions were performed in thermal cycler (Flexid Eppendorf Mastercycler, Germany) at a volume of 25µL including the following: 1.0µL of the extracted DNA, 12.5µL 2x My Taq Red Mix (BIOLINE), 0.8µL of each primer (10 pmol/µl; Macrogen, South Korea) and reached to the final volume by DNase/RNase free double distilled water. The PCR products were electrophoresed on 3% agarose gel and visualized by ultraviolet transillumination. 100 bp DNA Ladder (GDBio, China) was used as a marker. The functionality of primers was ensured via individual amplification.

**Table 2:** The sequence of the primers used for PCR amplification of virulence and carbapenemase-encoding genes

Target genes (bp)	Primer sequence (5`-3`)	Thermal cycler condition	References
Carbapenemase-encoding genes			
*blaKPC* (798 bp)	F: CGTCTAGTTCTGCTGTCTTG R: CTTGTCATCCTTGTTAGGCG	95 °C for 10 min, 30 cycles, 94 °C for 45 s, 54 °C for 45 s and 72 °C for 1 min; 72 °C for 10 min.	[27]
*blaNDM* (621 bp)	F: GGTTTGGCGATCTGGTTTTC R: CGGAATGGCTCATCACGATC		
*blaVIM* (390 bp)	F: GATGGTGTTTGGTCGCATA R: CGAATGCGCAGCACCAG		
*blaIMP* (232 bp)	F: GGAATAGAGTGGCTTAAYTCTC R: GGTTTAAYAAAACAACCACC		
*blaOXA-48* (438 bp)	F: GCGTGGTTAAGGATGAACAC R: CATCAAGTTCAACCCAACCG		
Virulence genes			
*clbA* (1311 bp)	F: CTAGATTATCCGTGGCGATTC R: CAGATACACAGATACCATTCA	95 °C for 10 min, 30 cycles, 94 °C for 45 s, 52 °C for 45 s and 72 °C for 1 min; 72 °C for 10 min.	[16] [28]
*clbB* (579 bp)	F: GATTTGGATACTGGCGATAACCG R: CCATTTCCCGTTTGAGCACAC		
*fimH* (688 bp)	F: ATGAACGCCTGGTCCTTTGC R: GCTGAACGCCTATCCCCTGC		
*mrkD* (240 bp)	F: CCACCAACTATTCCCTCGAA R: ATGGAACCCACATCGACATT		

### Statistical analysis

The statistical data were analyzed via the Chi-square test or Fisher’s exact test using SPSS version 18 software. The resulting data were considered significant if the *P*-value was < 0.05.

## Results

### Antimicrobial susceptibility test

**Fig. 1:**
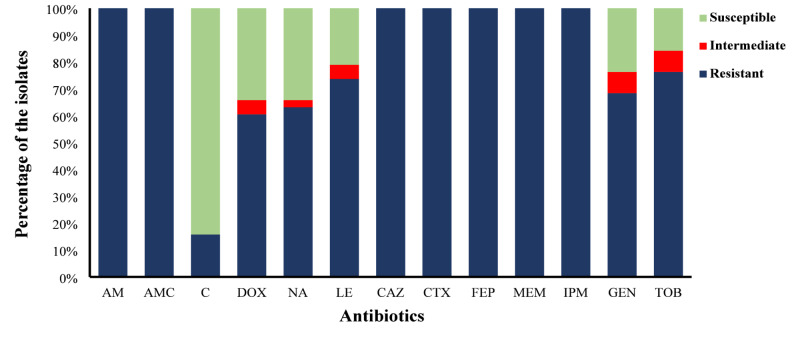
The antimicrobial susceptibility profile of CR-Kp isolates (*n* = 38). Each column represents an antibiotic, with colored segments indicating the percentage of isolates classified as resistant, intermediate, or susceptible. AM: Ampicillin; AMC: amoxicillin/clavulanate; C: Chloramphenicol; DOX: Doxycycline; NA: Nalidixic acid, LE: Levofloxacin; CAZ: Ceftazidime; CTX, Cefotaxime; FEP, Cefepime; MEM: Meropenem; IPM: Imipenem; GEN: Gentamicin; TOB: Tobramycin

The antibiogram profile showed that 44.2% (38/86) of *K. pneumoniae* isolates were resistant to meropenem and imipenem. All CR-Kp isolates exhibited 100% resistance to ampicillin, amoxicillin/clavulanate, and cephalosporins (ceftazidime, cefotaxime, cefepime). The next highest resistance was observed in the aminoglycoside class, including tobramycin (76.3%) and gentamicin (68.4%). Additionally, the resistance for fluoroquinolone class ranged from 73.7% (levofloxacin) to 63.2% (nalidixic acid), followed by doxycycline (60.5%). The lowest resistance rate (15.8%) was observed for chloramphenicol (Fig. 1).

### Phenotypic detection of carbapenemase production

It was observed that 92.1% (35/38) of CP-Kp isolates were positive by CarbaNP test. The positive results were observed within an incubation period ranging from 15 min to 2 h. Additionally, all isolates (38/38, 100%) were positive by mCIM test, and MBL-producing isolates were detected in 65.8% (25/38) of the isolates by eCIM test.

### Genotypic detection of carbapenemase genes

Consistent with the phenotypic results, the multiplex PCR assay estimated that only 44.2% (38/86) of *K. pneumoniae* harbored carbapenemase-encoding genes, either as a single gene or in combination. The PCR results showed that 71% of the isolates harbored *blaNDM*, while 36.8% of the isolates harbored *blaOXA-48*, and only 5.2% of the isolates harbored *blaKPC.* However, *blaIMP* and *blaVIM* genes were not detected. Furthermore, 13.15% of the isolates carried out both *blaNDM* and *blaOXA-48* genes.

**Fig. 2:**
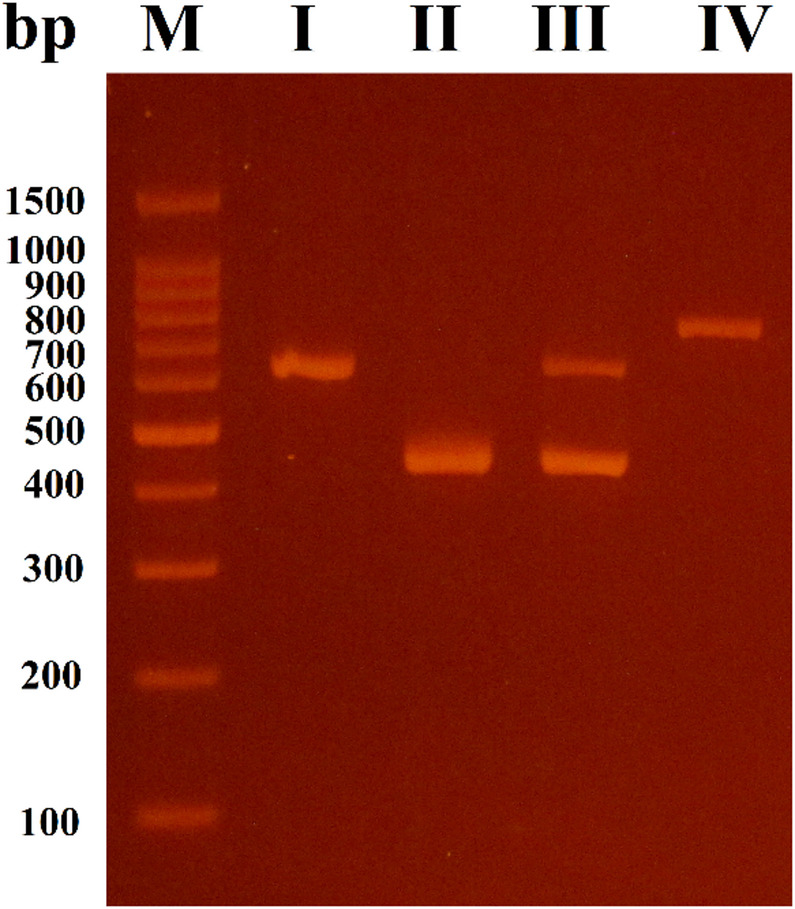
The agarose gel electrophoresis for the amplified multiplex PCR products of carbapenemase-encoding genes revealed four different patterns: I for *blaNDM* (621 bp); II for *blaOXA-48* (438 bp); III for *blaNDM* (621 bp) and *blaOXA-48* (438 bp); and IV for *blaKPC* (798 bp). M: 100 bp DNA ladder. Full-length gels are presented in Supplementary Fig. 2

The distribution of carbapenemase-encoding gene was observed in four different patterns as shown in Fig. 2. Specifically, pattern I had 22 isolates that harbored *blaNDM* as a single gene, II had 9 isolates that harbored *blaOXA-48* as a single gene, III had 5 isolates that co-harbored *blaNDM* and *blaOXA-48*, and finally pattern IV indicated the presence of only 2 isolates that harbored *blaKPC*.

### Correlation between phenotypic and genotypic detection of CP-Kp isolates

In the current study, all *K. pneumoniae* isolates that were phenotypically identified as carbapenemase producers via the mCIM test were positive for harboring one or more carbapenemase genes. While the CarbaNP test detected all isolates that harbored *blaNDM*, *blaKPC*, or co-harbored *blaNDM* and *blaOXA-48* and failed in the detection of 3 out of 9 isolates that harbored *blaOXA-48*. Additionally, the eCIM test detected the production of MBL in all isolates that carried out *blaNDM*. However, the isolates that co-harbored *blaNDM* and *blaOXA-48*, the eCIM identified only three isolates as MBL-producing and missed two isolates compared to the PCR results. Furthermore, the eCIM test accurately identified all isolates that harbored *blaOXA-48* or *blaKPC* as serine-producing, as shown in Table 3.

**Table 3:** The phenotypic and genotypic characterization of CP-Kp isolates (*n* = 38). Numbers in parentheses indicate the number of positive isolates

Genotypic characterization (*n*)	Phenotypic characterization		
	mCIM	CarbaNP	eCIM
*blaNDM* (22)	22	22	22
*blaOXA-48* (9)	9	6	0
*blaNDM + blaOXA-48* (5)	5	5	3
*blaKPC* (2)	2	2	0

### Phenotypic and genotypic association between the virulence factors and CP-Kp isolates

The biofilm characterization of CP-Kp isolates showed that 42.1% were moderate producers, 36.8% were strong producers, 13.1% were weak producers, and only 7.8% were non-biofilm producers. Additionally, the CN-Kp isolates revealed that 35.4% were non-biofilm producers, 25% were moderate producers, 20.8% were weak biofilm producers, and 18.7% were strong producers. Statistically, biofilm formation was significantly associated with CP-Kp isolates (Table 4).

**Table 4:** The association between carbapenemase phenotype and biofilm formation

Biofilm formation	Carbapenemase phenotype (n)		*P-*value
	CP-Kp (38)	CN-Kp (48)	
	n (%)	n (%)	
Strong	14 (36.8%)	9 (18.7%)	0.0069
Moderate	16 (42.1%)	12 (25.0%)	
Weak	5 (13.1%)	10 (20.8%)	
Non-biofilm	3 (7.8%)	17 (35.4%)	

**Fig. 3:**
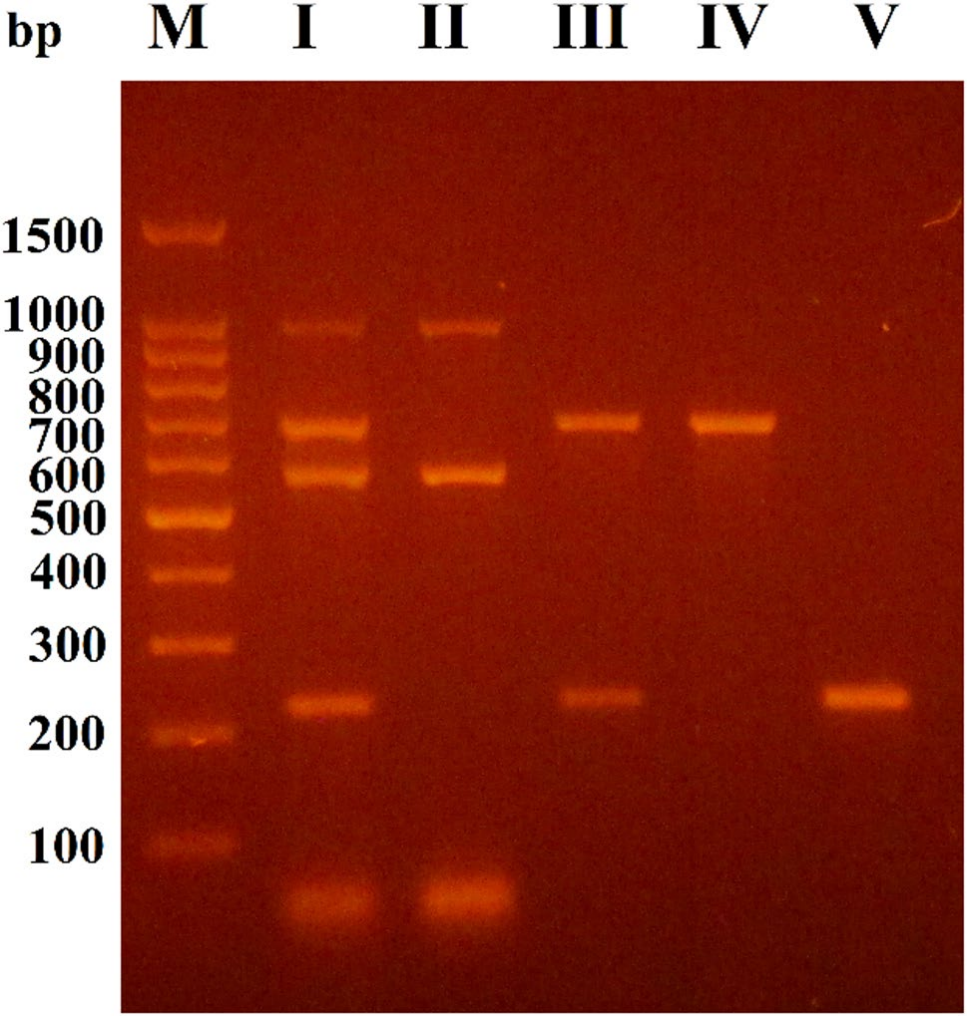
The agarose gel electrophoresis for the amplified multiplex PCR products of the virulence genes revealed five different patterns. I for *clbA* (1311 bp), *fimH* (688 bp), *clbB* (579 bp), and *mrkD* (240 bp); II for *clbA* (1311 bp) and *clbB (*579 bp); III for *fimH* (688 bp) and *mrkD* (240 bp); IV for *fimH* (688 bp); and V for *mrkD* (240 bp). M: 100 bp DNA ladder. Full-length gels are presented in Supplementary Fig. 3

The molecular detection of virulence genes indicated that *mrkD* was observed in 92.1% (35/38), while *fimH* was detected in 78.9% (30/38) of CP-Kp isolates. Only 13.2% (5/38) of CP-Kp isolates harbored colibactin-encoding genes (*clbA* and *clbB)*. Additionally, it was important to highlight that 20.9% (18/86) of *K. pneumoniae* isolates were *pks*^+^ isolates. Five different patterns were observed in the amplification of virulence genes among CP-Kp isolates: pattern I included 4 isolates that harbored all the virulence genes (*clbA*,* fimH*,* clbB*, and *mrkD*), while pattern II had only one isolate that carried out *clbA* and *clbB* genes. Furthermore, pattern III had 24 isolates that harbored both *mrkD* and *fimH* genes. Patterns IV and V demonstrated the presence of 2 isolates harboring the *fimH* gene and 7 harboring the *mrk*D gene, respectively, as shown in Fig. 3. A significant association was observed between CP-Kp isolates and the *mrkD* gene; in contrast, no significant correlation was detected between CP-Kp isolates and colibactin-encoding genes or the *fimH* gene (Table 5).

**Table 5:** Co-association between virulence genes and carbapenemase production

Virulence Genes		Carbapenemase production	
		-	+
*mrkD*	-	31	3
	+	17	35
	*P- value*	**< 0.0001**	
*fimH*	-	12	8
	+	36	30
	*P- value*	0.79	
*clbA*	-	35	33
	+	13	5
	*P-value*	0.18	
*clbB*	-	35	33
	**+**	13	5
	*P-value*	0.18	

The data were analyzed using Fisher’s exact test. *P-value* in bold is considered statistically significant. -: negative. +: positive

## Discussion

Despite global concern about controlling the emergence of AMR, it remains a critical issue threatening healthcare settings worldwide. Recently, there has been a significant concern about the high prevalence of CP-Kp isolates, resulting in limited treatment options, high mortality rates, and high cost due to the long period of hospitalization [29]. The current study reported that 44.2% of *K. pneumoniae* isolates were carbapenemase producers. In this regard, another study found that 58.42% of isolates were identified as CP-Kp [30]. In another study, carbapenemase producers were reported in a percentage of 33.3% among *K. pneumoniae* isolates [31]. Conversely, Ashour and El-Sharif [32] reported that 13.9% of *K. pneumoniae* isolates were identified as carbapenemase producers. The high incidence of carbapenemase producers over the recent decades could be linked to various factors, including the lack of compliance with infection control policies and the intensive use of carbapenems in the therapy strategy [33].

It was observed that the antibiogram profile of CP-Kp isolates aligned with the findings of El-Kholy et al. [34]. However, chloramphenicol exhibited the lowest resistance rate (15.8%); this was in contrast to Albasha et al. [35], who reported that 38% of CP-Kp isolates showed resistance to chloramphenicol. The disparity between the aforementioned results in resistance rates may be attributed to variations in geographical regions, the patient’s immune status, alterations in infection control policies, and inappropriate antimicrobial therapy.

Testing the phenotypic production of carbapenemases is a crucial step in the treatment to prevent the overuse of inappropriate antibiotics and support epidemiological research [36]. In this study, the CarbaNP test indicated that 92.1% of *K. pneumoniae* were carbapenemase producers. However, another study reported that 94.6% of *K. pneumoniae* isolates showed positive carbapenemase activity using the CarbaNP test [25]. In addition, the low sensitivity of the CarbaNP test is combined with *K. pneumoniae* isolates that harbored the *blaOXA-48*. This might be attributed to the low hydrolyzing activity of *blaOXA-48* [3].

The detection and distinguishing between MBL and serine carbapenemase-producing *K. pneumoniae* were employed using the mCIM/eCIM assay. All carbapenemase producers showed positive results with the mCIM test. In consistent with Ramadan et al. [37] and El-kholy et al. [34], the eCIM test accurately identified serine carbapenemases that harbored either *blaOXA-48* or *blaKPC*, and MBL-producing isolates that harbored *blaNDM*, but mis-distinguished the MBL production in 2 isolates that co-harbored *blaNDM* and *blaOXA-48.* The undetected MBL might be due to either a lack of gene expression or truncated genes, resulting in nonfunctional proteins [38].

Regarding the PCR results, the *blaNDM* gene was the most prevalent carbapenemase gene, followed by *blaOXA-48* and *blaKPC*. However, *blaVIM* and *blaIMP* genes were not detected. This prevalence aligns with Ramadan et al. [37]. Another study also reported the highly prevalent gene was *blaNDM*, followed by *blaOXA-48*, with the absence of *blaVIM*,* blaIMP*, or *blaKPC* [30]. In contrast, Taha et al. [39] found that *blaOXA-48* was the predominant gene, followed by *blaVIM*, *blaIMP*, *blaKPC*, and *blaNDM*. In this study, 13.15% of CP-Kp isolates co-harbored *blaNDM* and *blaOXA-48* genes, which is consistent with Kamalakar et al. [25], who reported a prevalence rate of 16.2%. Conversely, a previous study showed that 64.86% of the CP-Kp isolates harbored both *blaNDM* and *blaOXA-48* [40].

Interestingly, the capacity for biofilm formation differed significantly between CN-Kp and CP-Kp isolates, consistent with other literature [30, 41]. The high incidence of the biofilm phenotype among CP-Kp isolates may be attributed to the role of carbapenemase in stimulating the regulatory pathway of biofilm formation, in addition to enhancing the expression of biofilm-related genes [42]. Despite the previous statistical significance between biofilm formation and carbapenemase production, it does not reflect the actual clinical reality. This may be attributed to the detection of strong biofilm producers among CN-Kp isolates and an inability of some CP-Kp isolates to produce biofilm. Therefore, biofilm formation is not solely enhanced by carbapenemase production but appears to be a multifactorial process [30].

The current study recorded that 20.9% of all isolates of *K. pneumoniae* were *pks*^+^ isolates. However, another Egyptian study showed that 27.6% of the isolates were *pks*^+^ isolates [18]. Regarding the prevalence of colibactin genotoxin among CP-Kp isolates, it was observed that 13.2% of CP-Kp were *pks*^+^ isolates. Therefore, the co-association of colibactin-encoding genes and carbapenemase production was not significant. This also aligns with a previous study that indicated the prevalence of colibactin-encoding genes is low among the highly antimicrobial-resistant strains [43]. This could be attributed to the concept of minimizing the metabolic cost, as *pks*^+^ isolates tend to harbor highly pathogenic serovars and virulence genes, resulting in low drug resistance.

Our study also found that the *mrkD* and *fimH* genes were recorded among CP-Kp isolates in a percentage of 92.1% and 78.9%, respectively. Conversely, another study showed that 100% of CP-Kp isolates harbored *mrkD* and *fimH* genes [34]. Additionally, no significant correlation was observed between the existence of *fimH* and CP-Kp isolates, as type 1 fimbriae are typically found in 90% of *K. pneumoniae*, whether environmental or clinical [44]. Furthermore, it was found that the *mrkD* gene was significantly associated with CP-Kp isolates. However, another study indicated that there was no significant correlation between carbapenem producers and either the *mrkD* or *fimH* genes [45]. In the present study, the observed upregulation of the *mrkD* gene among CP-Kp isolates may represent a survival mechanism due to the environmental stress associated with carbapenemase production, as the *mrkD* gene plays a pivotal role in biofilm formation [46]. As biofilm formation is suggested to be a multifactorial process and not solely dependent on carbapenemase production, further studies are required to elucidate the underlying mechanisms linking carbapenemase production and biofilm-related genes.

## Conclusion

The current study highlights important insights into the association between carbapenemase production and virulence factors in carbapenem-resistant *K. pneumoniae* clinical isolates. The emergence of such resistant bacteria, coupled with high virulence potential, poses a significant therapeutic challenge. To address this challenge, ongoing monitoring of antimicrobial resistance and virulence determinants, strict infection control policies, and accurate laboratory diagnosis of resistant pathogens to inform appropriate treatment strategies are highly recommended. Furthermore, the occurrence of colibactin genotoxin among CP-Kp isolates presents an alarming scenario due to the potential pairing of genotoxicity and high drug resistance in the future, which requires further studies into their epidemiological characteristics.

## Supplementary Information

Supplementary Material 1.

Supplementary Material 2.

## Data Availability

The data in the current study are available via the corresponding author upon a reasonable request.

## Abbreviations

AMR: Antimicrobial Resistance
ESBLs: Extended spectrum β-Lactamases
CP-Kp: Carbapenemase-producing *Klebsiella pneumoniae*
CR-Kp: Carbapenem-resistant *Klebsiella pneumoniae*
CN-Kp: Carbapenemase non-producing *Klebsiella pneumoniae*
KPC: *Klebsiella pneumoniae* carbapenemase
MBLs: Metallo-β-lactamases
IMP: Imipenemase
NDM: New Delhi metallo-β lactamase
VIM: Verona integron-encoded metallo-β-lactamase
OXA: Oxacillinases
CLSI: Clinical and Laboratory Standards Institute
TCP: Tissue Culture Plate
TSB: Tryptic soy broth
PCR: Polymerase chain reaction
EDTA: Ethylenediaminetetraacetic acid
