# Supplementary material for: Phenotypic and genotypic characterization of carbapenem resistant Klebsiella pneumoniae clinical isolates from Damietta, Egypt
Source: BMC Microbiol. 2026 Feb 27;26:306. doi: 10.1186/s12866-026-04797-z (PMC13049837; doi:10.1186/s12866-026-04797-z)
Supplement: Supplementary file 1 — Supplementary Material 1. [file 12866_2026_4797_MOESM1_ESM.docx]

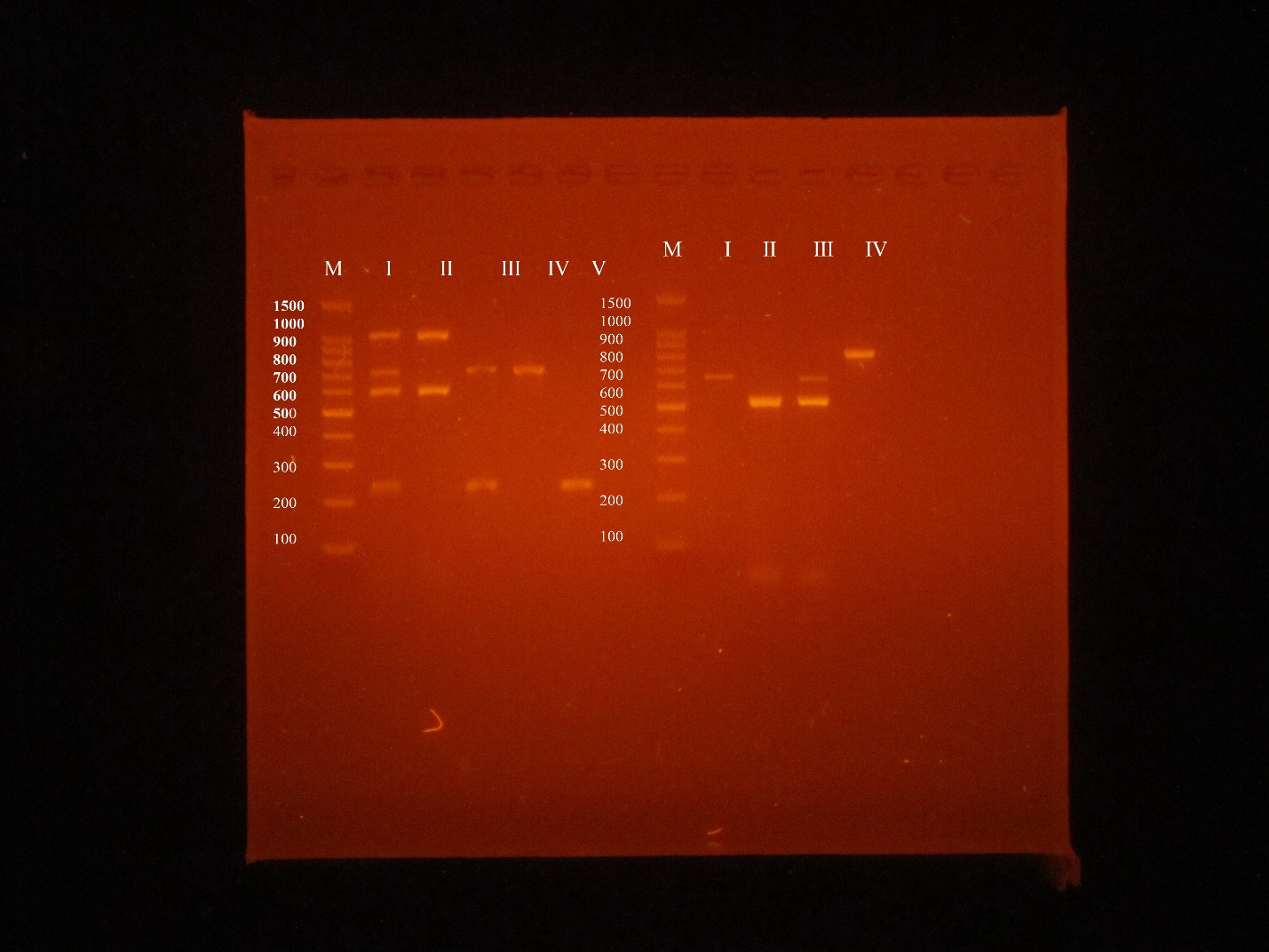


The figure showed the electrophoresis of the multiplex products of carbapenemase encoding genes (I, II, III, IV) and virulence genes (I, II, III, IV, V). M: 100bp DNA ladder. The figure is clearly defined edges


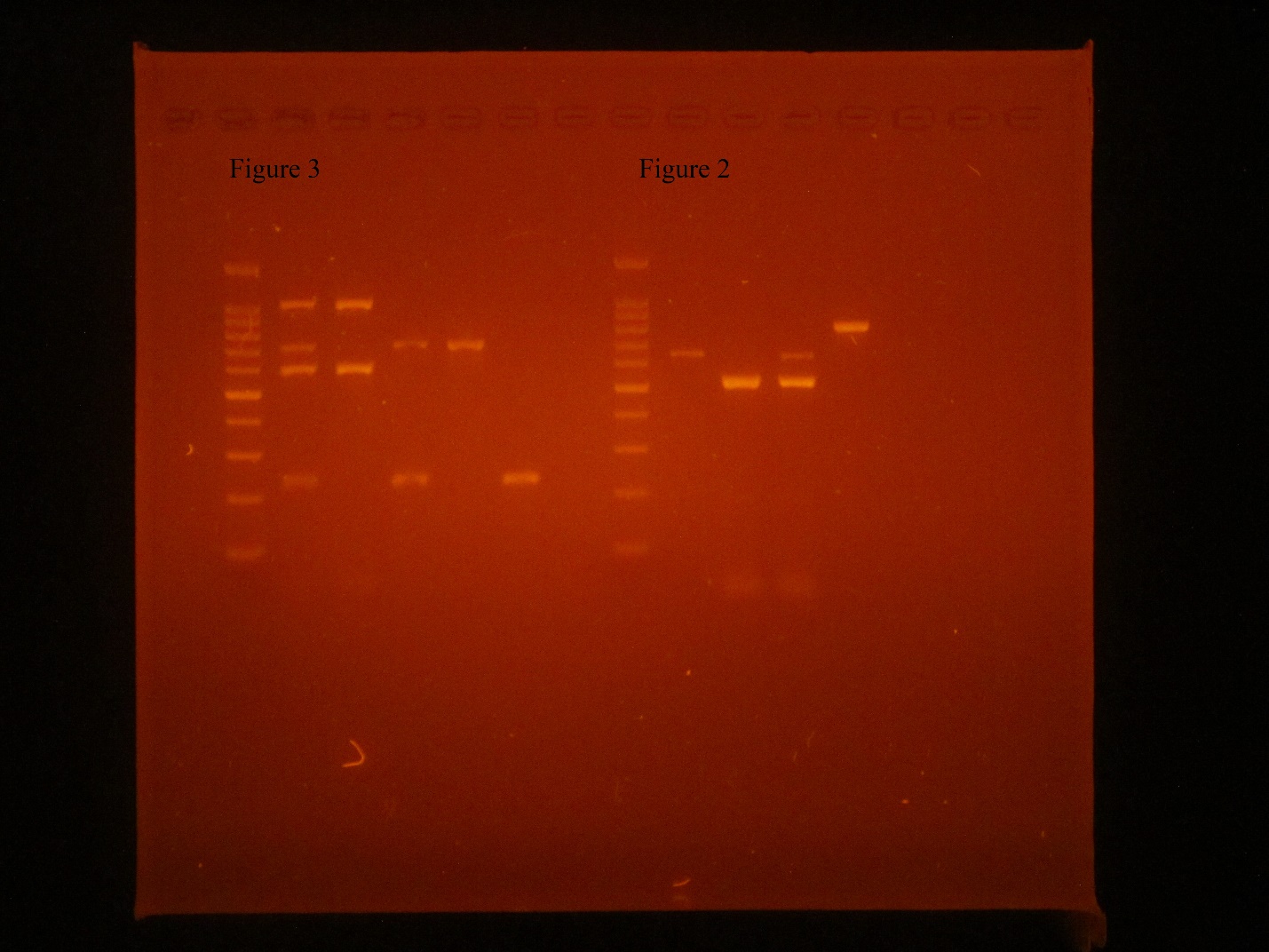


The figure showed the electrophoresis of the multiplex products of carbapenemase encoding genes (figure 2) and virulence genes (figure 3). The figure is clearly defined edges


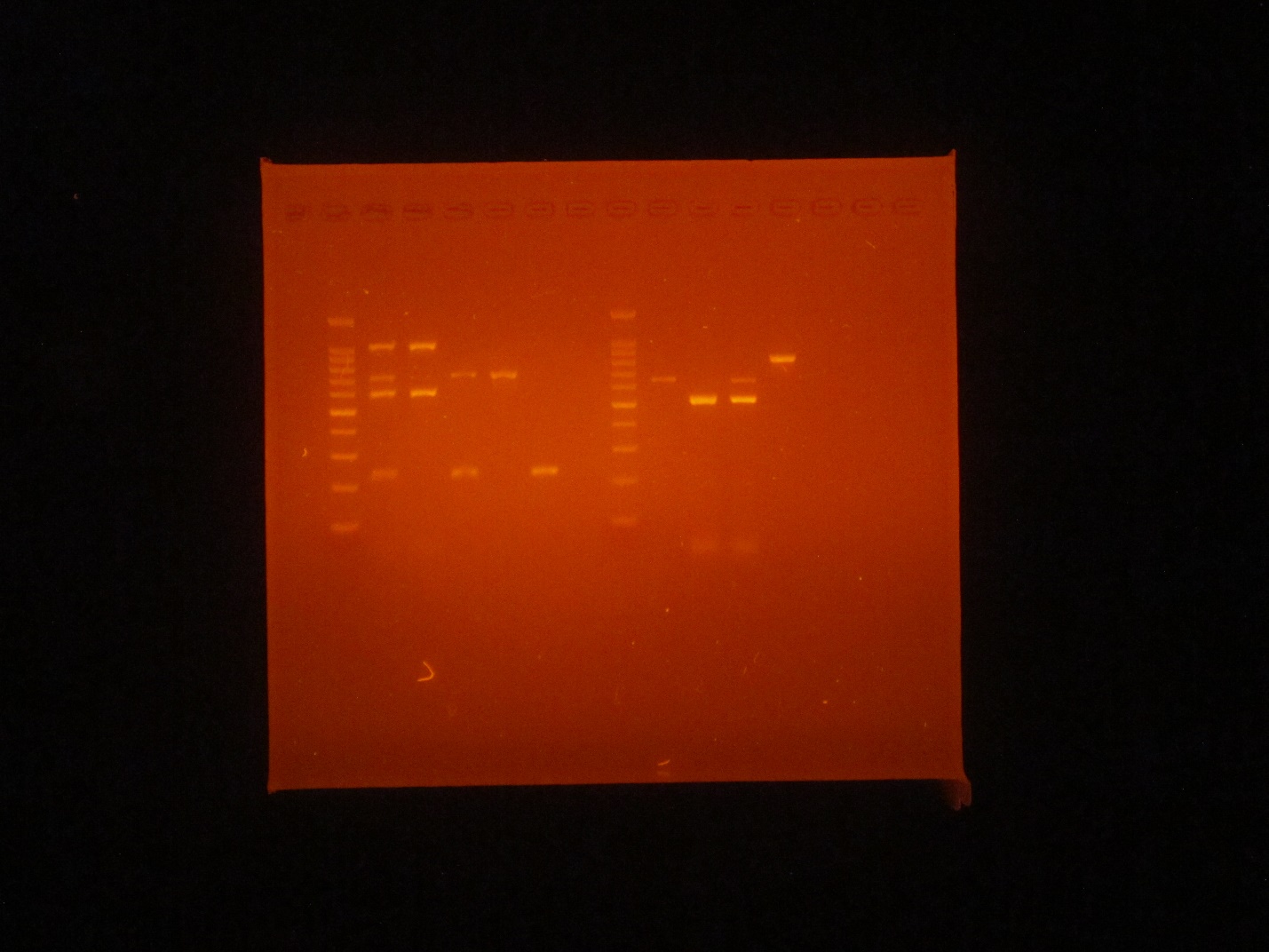


The figure represented another replica for the electrophoresis of the multiplex products of carbapenemase encoding genes (figure 2) and virulence genes (figure 3). The figure is clearly defined edges
