# Supplementary material for: Phenotypic and genotypic characterization of carbapenem resistant Klebsiella pneumoniae clinical isolates from Damietta, Egypt
Source: BMC Microbiol. 2026 Feb 27;26:306. doi: 10.1186/s12866-026-04797-z (PMC13049837; doi:10.1186/s12866-026-04797-z)
Supplement: Supplementary file 2 — Supplementary Material 2. [file 12866_2026_4797_MOESM2_ESM.docx]

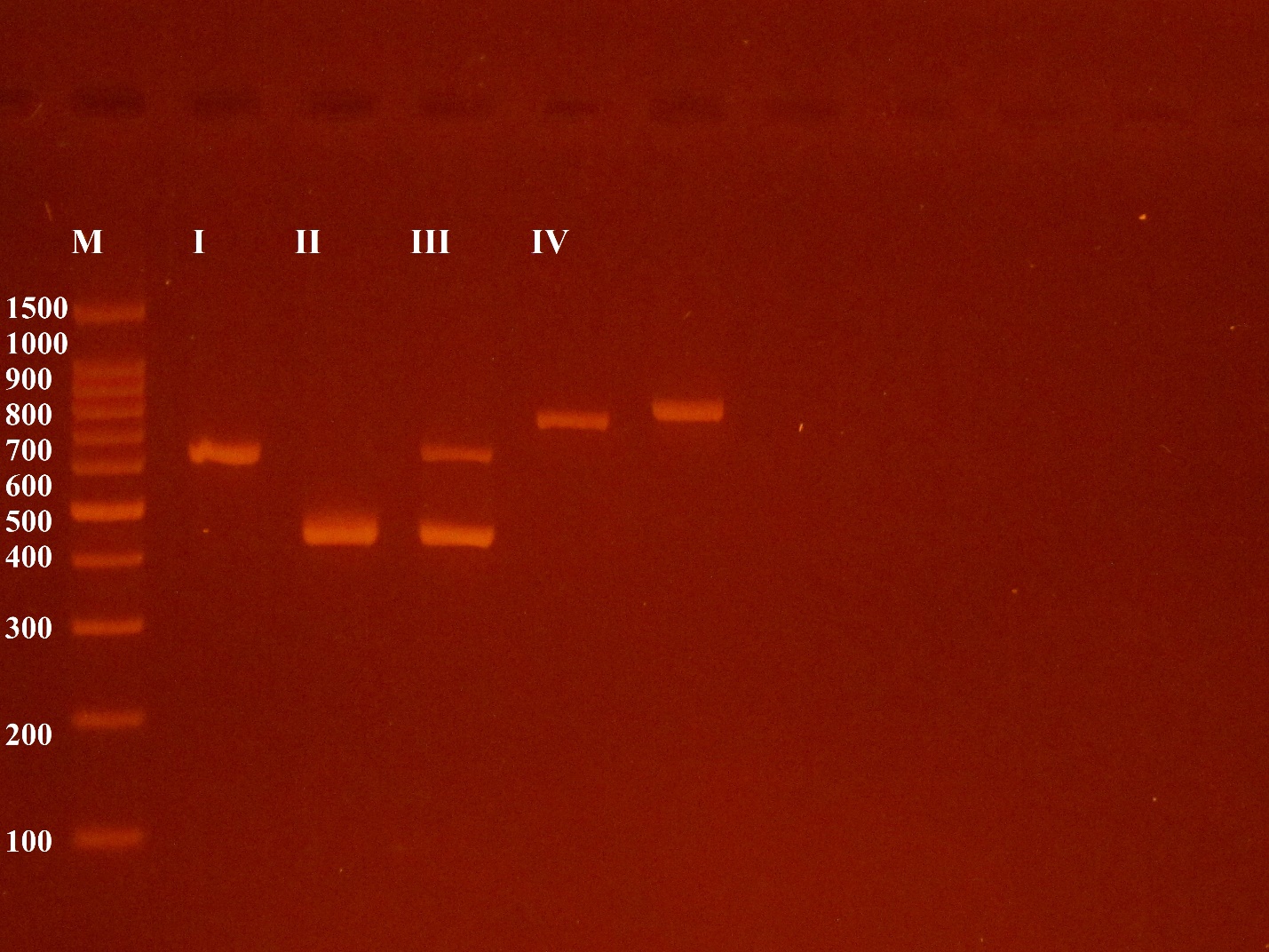


Fig. 2 The Full-length gel for the amplified multiplex PCR products of carbapenemase encoding genes revealed four different patterns (I, II, III, IV), M: 100bp DNA ladder. This gel was used for the Figure panel.


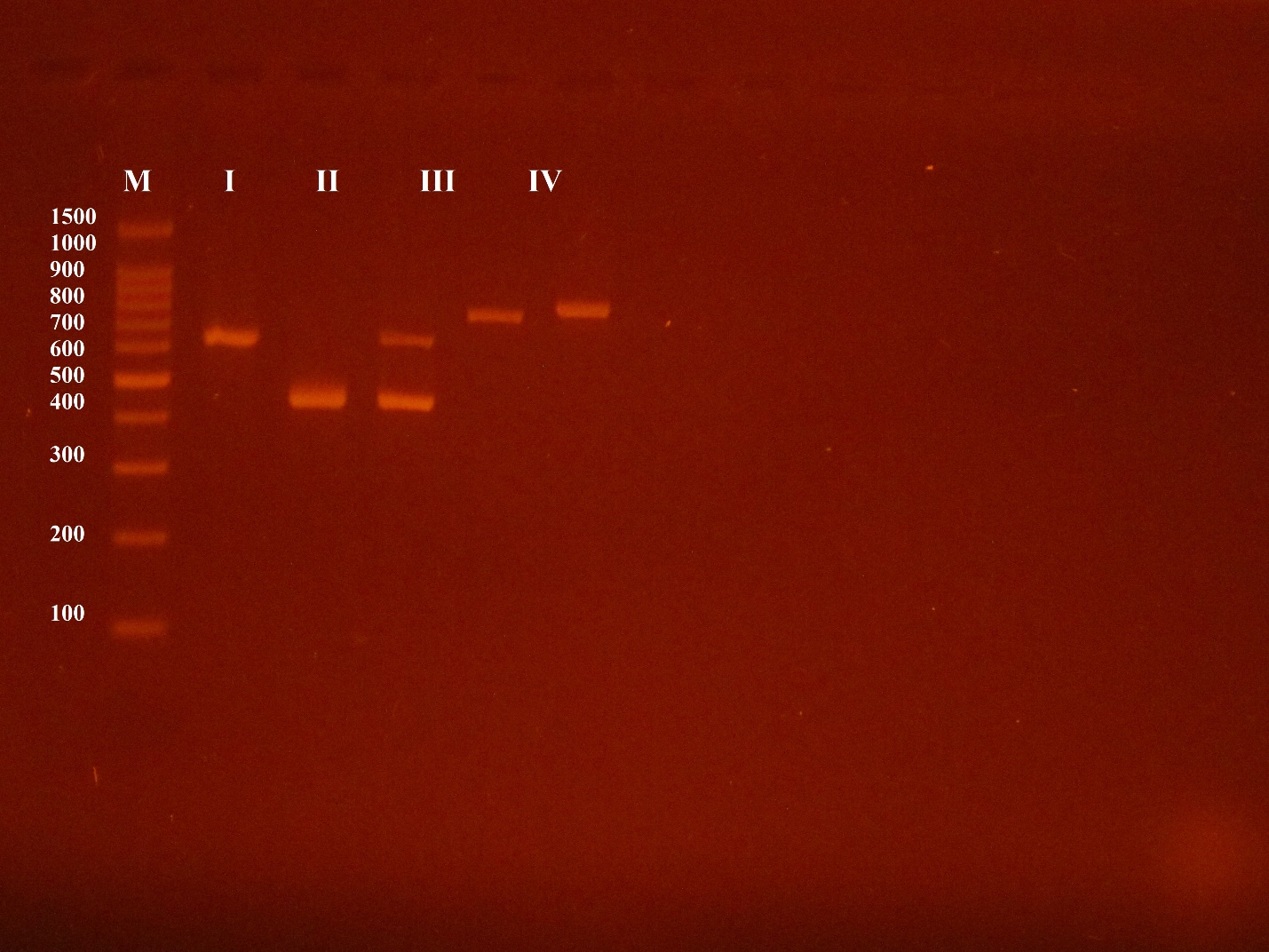


Fig. 2 The replicate of the Full-length gel for the amplified multiplex PCR products of carbapenemase encoding genes revealed four different patterns (I, II, III, IV), M: 100bp DNA ladder.


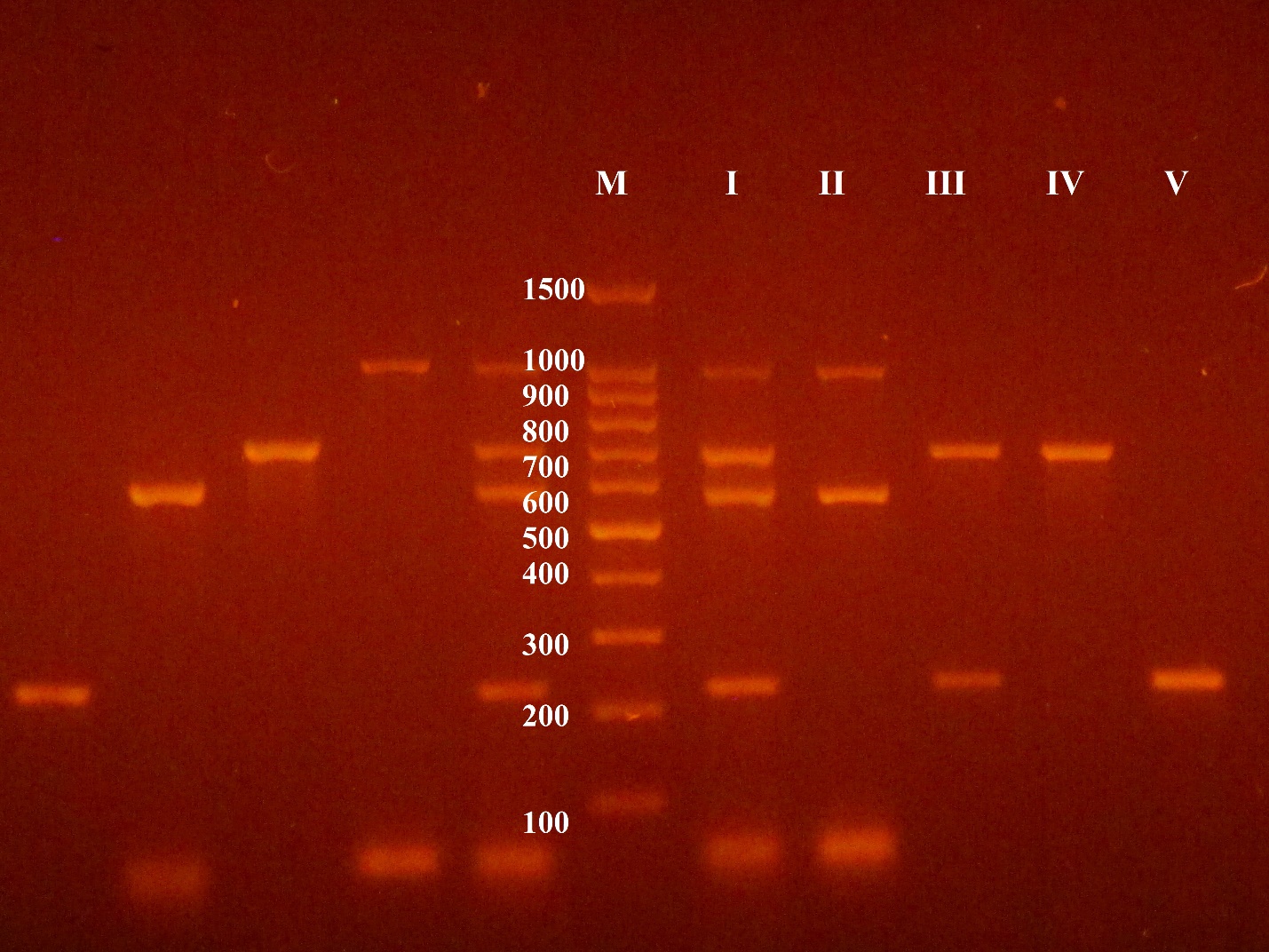


Fig.3 The Full-length gel for the amplified multiplex PCR products of the virulence genes revealed five different patterns (I, II, III, IV, V). M: 100bp DNA ladder. This gel was used for the Figure panel.


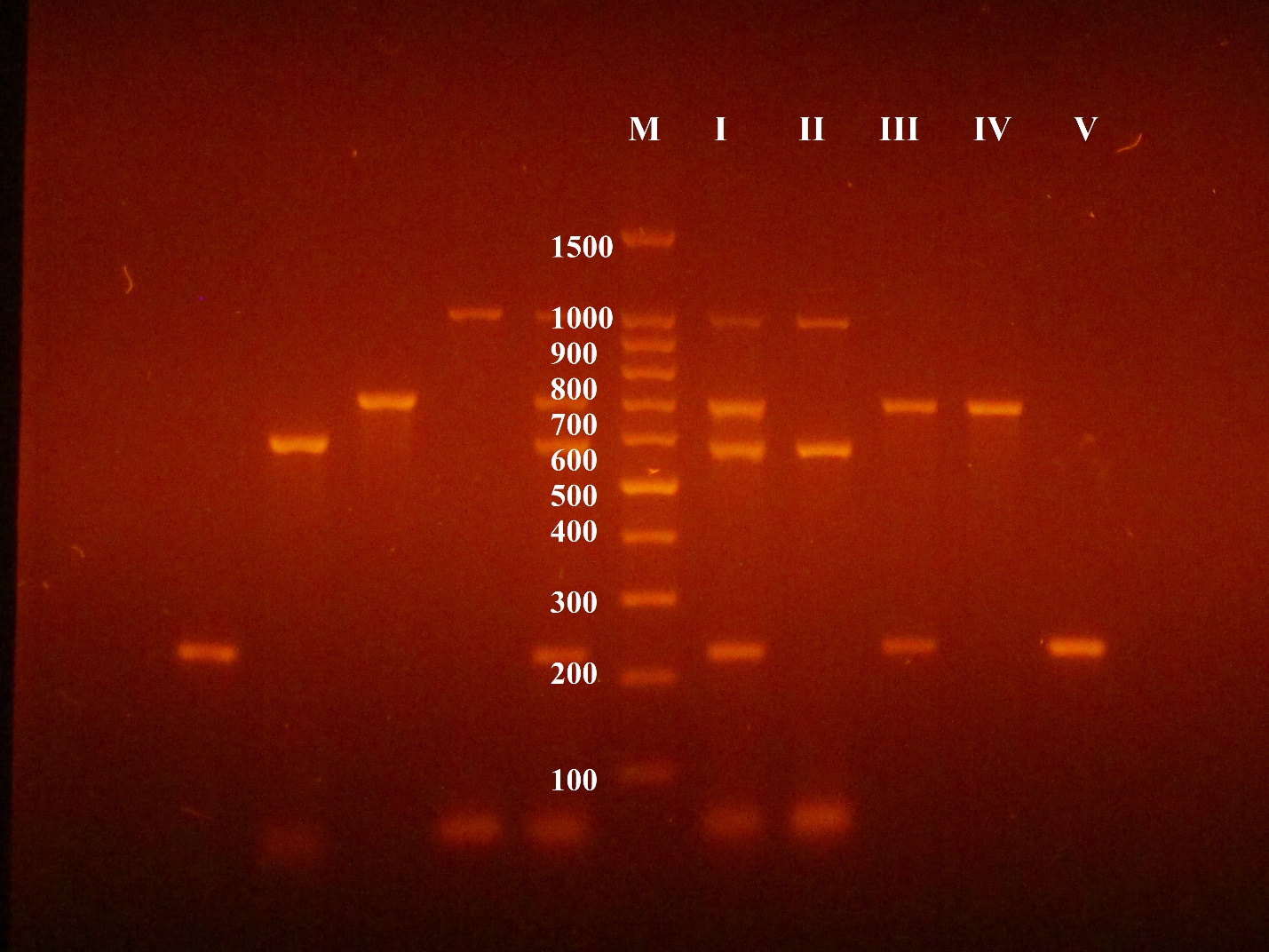


Fig.3 The replicate of the Full-length gel for the amplified multiplex PCR products of the virulence genes revealed five different patterns (I, II, III, IV, V). M: 100bp DNA ladder.
